# Keep Your TEMPO Up: Nitroxide Radicals as Sensors of Intermolecular Interactions

**DOI:** 10.3390/molecules29215032

**Published:** 2024-10-24

**Authors:** Ilya G. Shenderovich

**Affiliations:** Institute of Organic Chemistry, University of Regensburg, Universitaetstrasse 31, 93053 Regensburg, Germany; ilya.shenderovich@ur.de

**Keywords:** TEMPO, EPR, hyperfine coupling constant, hydrogen bonding, non-covalent interactions

## Abstract

This study examines experimental data on the influence of the surrounding medium and non-covalent interactions on the isotropic hyperfine coupling constant, A_iso_(^14^N), of the stable nitroxide radical 2,2,6,6-Tetramethylpiperidin-1-yl)oxyl (TEMPO) in solution. The data were used to identify a density functional theory functional/basis set combination that accurately reproduces the experimental A_iso_(^14^N) values. The variations in A_iso_(^14^N) due to external factors are two orders of magnitude greater than the accuracy of its experimental measurements, making A_iso_(^14^N) a highly sensitive experimental probe for quantifying these effects. Additionally, it was found that the proton-accepting ability of the N-O^•^ moiety in TEMPO resembles that of the P=O moiety, enabling the simultaneous formation of two equally strong hydrogen bonds.

## 1. Introduction

The interaction of molecules with nitroxide radicals induces several characteristic spectral changes. This interaction affects the chemical shifts and relaxation times of the nuclei, as well as the appearance of the unpaired electron spin density on these nuclei. The most important actual application of stable radicals is the technique of dynamic nuclear polarization (DNP) [1]. This technique has many applications for molecular systems of varying complexity [2,3,4,5,6].

Paramagnetic shifts induced by nitroxide radicals in surrounding molecules depend on their interaction [7]. The most commonly studied nucleus for such analysis has been the ^13^C nucleus [8,9,10,11]. The method of ^13^C NMR of TEMPO-functionalized polymers has been used to study the thermoreversible phase transition of aqueous solutions of these polymers [12]. Effects have also been observed on the ^1^H, ^19^F, and ^15^N nuclei [13]. The method of ^1^H NMR was used to study the pressure effect on intermolecular interactions with nitroxide radicals [14].

Intermolecular interactions with nitroxide radicals can be characterized by studying their effect on the relaxation times of their diamagnetic partners [15,16,17,18,19,20,21,22,23]. The most definitive confirmation of complex formation between diamagnetic molecules and nitroxide radicals is the appearance of unpaired electron spin density on the nuclei of these molecules [24,25]. Nitroxide radicals act as proton acceptors, with hydrogen bonding being the most prominent mechanism for the formation of such complexes [26,27]. Complexes with alcohols [28,29,30,31,32,33,34], anilines [35,36], amines [24], acids [37,38], water [39], and phenols [40,41] are among the best studied.

On the other hand, intermolecular interactions cause two characteristic spectral changes in the nitroxide radicals themselves. These interactions manifest in changes in the isotropic hyperfine coupling constant A_iso_(^14^N) [42,43] and variations in the relative linewidths of the components of the radical EPR signal. This difference in linewidth depends on the rate of rotational diffusion of the radical and can be used to study the physical properties of viscous liquids [44], ionic liquids [45,46], polymers [47], micelles [48,49], and inclusion complexes [50].

All the above parameters can be used either individually or in combination when utilizing radicals as probes to study complex molecular systems. However, in this publication, only the effect of molecular complexation on A_iso_(^14^N) will be considered and discussed. This parameter is easy to measure, and, apparently, its changes are detectable even in cases of weak and transient interactions [51]. A_iso_(^14^N) can be used to analyze steric effects on the accessibility of active sites [52,53,54,55,56] in a wide variety of weak proton donors and local micropolarity [57,58]. Such an analysis may provide a better understanding of the factors affecting the reactivity of these centers, whether through intramolecular interactions [59,60] or external influences [61,62]. Other studies [63,64] suggest that A_iso_(^14^N) can also serve as a sensor for halogen bond properties [65,66].

**Figure 1:**
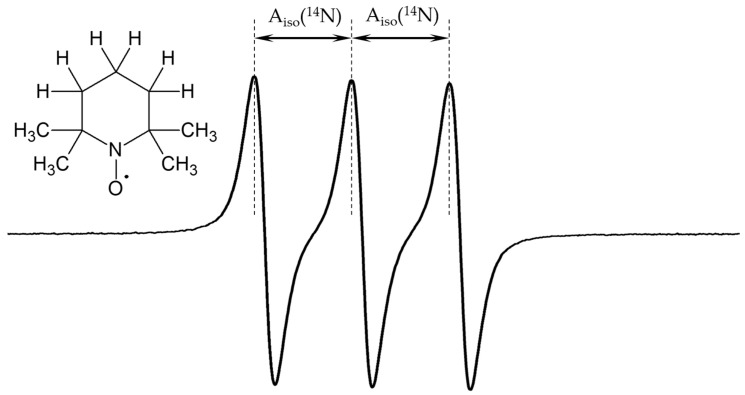
EPR spectrum of 5 × 10^−4^ M (2,2,6,6-Tetramethylpiperidin-1-yl)oxyl (TEMPO) in toluene at 300 K. A_iso_(^14^N) = 1.55 mT, g = 2.0023.

The objectives of this study are as follows: (i) to identify a density functional theory (DFT) functional/basis set pair that accurately reproduces the experimental values of A_iso_(^14^N) in aprotic solvents, which will then be used to interpret A_iso_(^14^N) observed experimentally (ii) in water and alcohols, and (iii) in toluene solutions of phenols; (iv) to theoretically estimate the maximum possible value of A_iso_(^14^N); and (v) to theoretically estimate the effect of halogen bonds on A_iso_(^14^N). The radical (2,2,6,6-Tetramethylpiperidin-1-yl)oxyl (TEMPO) will be used as the model nitroxide radical. Figure 1 shows the molecular structure of this stable radical and its EPR spectrum in toluene. Wherever possible, calculated A_iso_(^14^N) values are compared with the corresponding experimental values available in the literature.

## 2. Results and Discussion

### 2.1. The Best Functional/Basis Set Pair for Calculating A_iso_(^14^N)

Different DFT functional/basis set pairs have been tested in the past for calculations related to radicals. [67,68,69]. Here, several DFT functionals and basis sets were evaluated to calculate A_iso_(^14^N) for TEMPO in toluene. Both the geometry of TEMPO and its A_iso_(^14^N) were calculated using the polarizable continuum model approximation (PCM) with SCRF=(Solvent=toluene). The observed experimental value of A_iso_(^14^N) decreases with increasing radical concentration [35,70]. Therefore, reliable calculated values of A_iso_(^14^N) should equal or exceed the experimental value. Table 1 reports the calculated values of A_iso_(^14^N) for selected DFT functional/basis set pairs.

The uPBE1PBE/LanL2DZ, uHSEH1PBE/LanL2DZ, and uHSEH1PBE/LanL2DZ pairs give results that are very close to the experimental value of 1.551 mT. However, in all cases, the calculated values are slightly lower than the experimental value. A possible reason for this deviation is that the effect of the reaction field created by the polarization of the surrounding dielectric cannot be correctly modeled within the PCM approach [58,71]. This can be addressed using a fictitious external electric field [71,72,73]. However, since the required field strength is solvent-dependent and cannot be estimated a priori, further calculations will be performed using the PCM approximation. If this explanation is correct, then the deviation between the theoretical and experimental values should increase with increasing solvent polarity.

### 2.2. Aprotic Solvents

Table 2 reports the available experimental values of A_iso_(^14^N) for TEMPO in aprotic solvents. For some of these solvents, A_iso_(^14^N) was calculated in the uPBE1PBE/LanL2D approximation. Both the geometry of TEMPO and its A_iso_(^14^N) were calculated in the PCM approximation using the solvent under study. In most cases, the calculated values are lower than the experimental ones. However, the deviations are not significant. The obtained data do not enable one to confirm or refute the assumption above that the deviation between the theoretical and experimental values increases with increasing solvent polarity. Nevertheless, the calculations correctly reproduce the trend for A_iso_(^14^N) to increase with increasing solvent polarity. Notably, these changes are substantial enough to track even small variations in polarity. The experimental measurement error is on the order of 0.001 mT, and the absolute value changes by 0.1 mT when transitioning from hexane to (methanesulfinyl)methane (DMSO).

### 2.3. Protic Solvents

**Figure 2:**
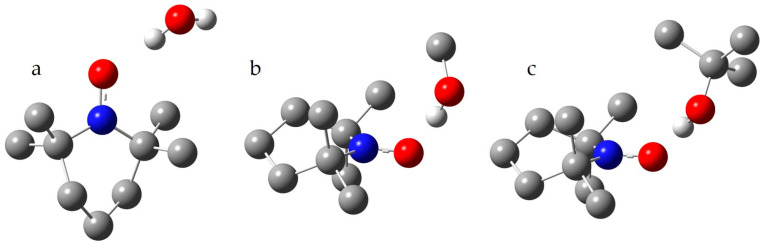
The model structures of the selected 1:1 complexes of TEMPO with (**a**) water, (**b**) methanol, and (**c**) 2-methylpropan-2-ol. Oxygen atoms are represented in red, nitrogen atoms in blue, carbon atoms in grey, and exchangeable protons in white.

Table 3 reports the available experimental values of A_iso_(^14^N) for TEMPO in protic solvents. For some of these solvents, A_iso_(^14^N) in 1:1 complexes of TEMPO and a solvent molecule was calculated in the uPBE1PBE/LanL2D approximation. Both the geometry of the complexes and A_iso_(^14^N) were calculated in the PCM approximation using the solvent under study. The structures of some of these complexes are shown in Figure 2.

Figure 2a shows the structure of the complex with a water molecule. The calculated A_iso_(^14^N) value is lower than the experimental A_iso_(^14^N) value in water. There are several possible reasons for this. First, the effect of the reaction field created by the polarization of the surrounding dielectric cannot be correctly modeled within the PCM approach [71,72,73]. Second, the proton-donating ability of a water molecule in water is greater than that of a single water molecule [80]. Given these limitations, the calculation result can be considered satisfactory and unequivocally indicates that the oxygen atom of TEMPO is involved in the hydrogen bond network of water. Note that the calculated A_iso_(^14^N) value in the PCM SCRF=(solvent=water) approximation is only 1.5984 mT. Therefore, the hydrogen bond leads to an increase in A_iso_(^14^N) by more than 0.12 mT.

Figure 2b,c shows the structures of two 1:1 complexes of TEMPO with alcohol molecules. For all studied alcohols, the calculated A_iso_(^14^N) values in such complexes are similar to those with water but significantly exceed the available experimental values of A_iso_(^14^N) in these alcohols, as seen in Table 3. On the other hand, these experimental values significantly exceed the calculated A_iso_(^14^N) values in the PCM SCRF=(solvent=alcohol) approximation. The hydrogen bonds in these 1:1 complexes lead to an increase in A_iso_(^14^N) by more than 0.13 mT. The observed difference between the calculated and experimental values indicates that in alcohols, TEMPO may or may not be hydrogen-bonded. Greatly simplifying the real situation and assuming that there is an equilibrium between TEMPO molecules that do not participate in any way in hydrogen bonding and TEMPO molecules forming the 1:1 complexes of optimal geometry with the alcohol molecules, one can write the following: A_iso_(^14^N)^exp^ = x_H_∙A_iso_(^14^N)^H^ + x_f_∙A_iso_(^14^N)^f^, where A_iso_(^14^N)^exp^, A_iso_(^14^N)^H^, and A_iso_(^14^N)^f^ stand for the observed experimental value, the calculated value in the 1:1 complex, and the calculated value for TEMPO in the PCM approximation, respectively. x_H_ and x_f_ are the mole fractions of the hydrogen-bonded and free TEMPO molecules, respectively, and x_H_ + x_f_ = 1. Thus, x_H_ = (A_iso_(^14^N)^exp^ − A_iso_(^14^N)^f^)/(A_iso_(^14^N)^H^ − A_iso_(^14^N)^f^). For TEMPO in methanol, x_H_ = (1.63 − 1.5933)/(1.7371 − 1.5933) ≈ 0.25. For TEMPO in 2-methylpropan-2-ol, x_H_ ≈ 0.15. Of course, these mole fractions must largely depend on the proton-donating ability of the alcohol molecule and the temperature.

### 2.4. Interaction of TEMPO with Phenols in Toluene

**Figure 3:**
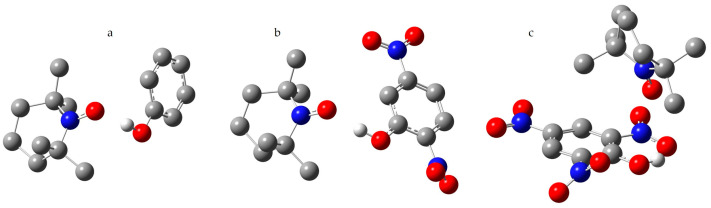
The model structures of the 1:1 complexes of TEMPO with (**a**) phenol, (**b**) 2,5-dinitrophenol, and (**c**) 2,4,6-trinitrophenol. Oxygen atoms are represented in red, nitrogen atoms in blue, carbon atoms in grey, and exchangeable protons in white.

Table 4 presents the available experimental values of A_iso_(^14^N) for TEMPO in solutions of phenols in toluene. For some of these phenols, A_iso_(^14^N) in 1:1 complexes of TEMPO and a phenol molecule was calculated in the uPBE1PBE/LanL2D approximation. Both the geometry of the complexes and A_iso_(^14^N) were calculated in the PCM approximation in toluene. The structures of these complexes are shown in Figure 3.

Phenol and 4-nitrophenol form with TEMPO hydrogen-bonded complexes, as seen in Figure 3a. The mole fractions of these complexes in the studied solution can be estimated using x_H_ = (A_iso_(^14^N)^exp^ − A_iso_(^14^N)^Tol^)/(A_iso_(^14^N)^H^ − A_iso_(^14^N)^Tol^), where A_iso_(^14^N)^Tol^ stands for the experimental value in toluene, 1.551 mT [52]. x_H_ depends on the phenol concentration. For concentrations of 0.01 M and 0.05 M, x_H_ is approximately 0.07 and 0.29 for phenol and 0.19 and 0.35 for 4-nitrophenol, respectively.

The hydrogen bond with 2,5-dinitrophenol should be even stronger, as seen in Table 4 and Figure 3b. However, the estimated values of x_H_ are only about 0.02 and 0.09 for concentrations 0.01 M and 0.05 M, respectively. This indicates that the presence of a nitro group in the *ortho* position greatly hinders the formation of the complex. If both *ortho* positions are occupied by nitro groups, 2,4,6-trinitrophenol, the hydroxyl group prefers to form an intramolecular hydrogen bond with one of them, as seen in Figure 3c. The calculated value of A_iso_(^14^N) in such a complex is low, as seen in Table 4. The absence of hydrogen bonding between 2,4,6-trinitrophenol and TEMPO in toluene is also confirmed by the low experimental value of A_iso_(^14^N). The same is true for 2,6-di-*tert*-butylphenol, as seen in Table 4. Therefore, *tert*-butyl groups at the *ortho* positions block the proton-donating ability of phenol just as they block the proton-accepting ability of pyridine [81].

Note that with a 5-fold increase in phenol concentration, the increase in x_H_ values depends on the type of phenol. For phenol, x_H_ increases 4-fold, for 4-nitrophenol it doubles, and for 2,5-dinitrophenol, it increases 5-fold. One can speculate that these changes reflect the instantaneous concentration of the TEMPO/phenol hydrogen-bonded complexes of different geometry in solutions at phenol concentrations of 0.01 M. Phenol is a weak proton donor, resulting in short lifetimes for its hydrogen-bonded complexes at room temperature. Consequently, most TEMPO molecules remain free when the phenol concentration is low. 2,5-dinitrophenol is a stronger proton donor, but the lifetime of its hydrogen-bonded complexes is limited by steric interference from its ortho substituent. Therefore, most TEMPO molecules remain free in this case, as well when phenol concentration is low. For both phenols, the energy gain from hydrogen bonding is offset by entropy losses. When most TEMPO molecules in solution remain free, a 5-fold increase in phenol concentration leads to a 5-fold increase in the concentration of short-living hydrogen-bonded complexes. In contrast, 4-nitrophenol is a strong proton donor with no steric hindrances to its hydrogen bonding, so the instantaneous concentration of TEMPO/4-nitrophenol hydrogen-bonded complexes is already significant at a phenol concentration of 0.01 M. In this case, an increase in the phenol concentration causes only a limited increase in the instantaneous concentration of the hydrogen-bonded complexes.

### 2.5. Interaction of TEMPO with Strong Proton Donors

What is the largest possible value for A_iso_(^14^N) of TEMPO? The largest experimentally measured values are around 2.18−2.19 mT [76,78], attributed to TEMPO-H^+^. Strong acids can cause chemical degradation of the nitroxide paramagnetic center [82,83,84,85], so the actual existence of the complexes discussed below cannot be confirmed here. The current goal is to estimate the Aiso(¹⁴N) values that can be expected for complexes between TEMPO and strong proton donors.

The calculated A_iso_(^14^N) value for the TEMPO-H^+^ cation is 2.42 mT, as seen in Table 5, with both its geometry and A_iso_(^14^N) value calculated using the PCM approximation in water. The orientation of proton donors relative to the N-O^•^ moiety of TEMPO, as shown in Figure 2 and Figure 3, suggests that its proton-accepting ability may be similar to the P=O moiety. This allows for the simultaneous formation of two hydrogen bonds of equal energy [86,87]. This feature of the P=O moiety significantly influences the structure of its hydrogen-bonded adducts [88,89,90,91,92]. The calculated A_iso_(^14^N) for the TEMPO-2H^2+^ cation is 2.45 mT, as seen in Table 5. Both its geometry and A_iso_(^14^N) value were calculated using the PCM approximation in water.

**Figure 4:**
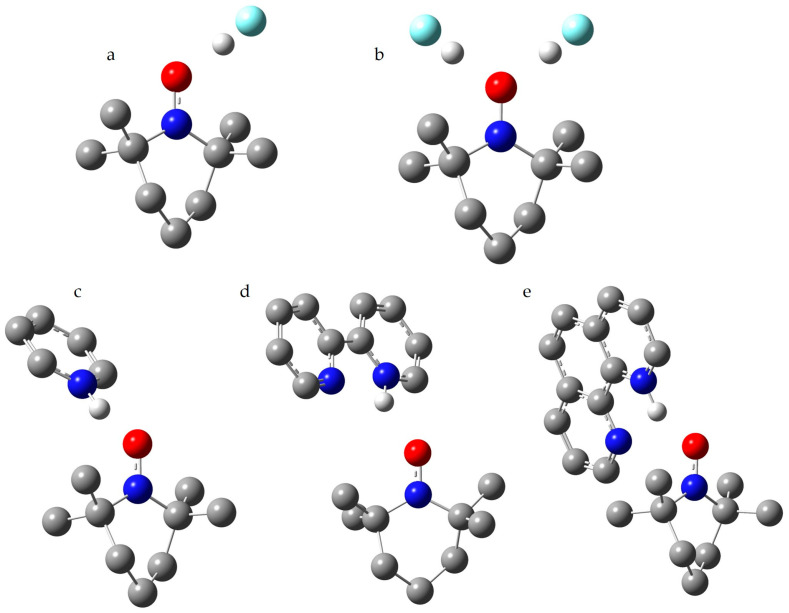
The model structures of the complexes of TEMPO with strong proton donors. (**a**) Hydrogen fluoride. (**b**) Two molecules of hydrogen fluoride. (**c**) Pyridine-H^+^ cation. (**d**) 2,2′-bipyridine-H^+^ cation. (**e**) 1,10-phenanthroline-H^+^ cation. Oxygen atoms are represented in red, nitrogen atoms in blue, carbon atoms in grey, fluorine atoms in cyan, and exchangeable protons in white.

To analyze the difference between TEMPO adducts with one and two hydrogen bond donors, its complexes with hydrogen fluoride (HF) were examined. Figure 4a,b shows their structures. Both the geometry of these adducts and their A_iso_(^14^N) value were calculated using the PCM approximation in water. Unlike the TEMPO cations discussed earlier, there is a significant difference in A_iso_(^14^N) for these adducts, as seen in Table 5. The formation of the second hydrogen bond in the TEMPO…2HF adduct leads to a lengthening of the O…H distances. Is the formation of two hydrogen bonds energetically favorable? The hydrogen bond energy in these adducts can be defined as the difference between (i) the sum of the electronic energies of a noninteracting TEMPO molecule and one or two noninteracting HF molecules, and (ii) the electronic energy of the TEMPO…HF or TEMPO…2HF adducts, all calculated using the PCM approximation in water. For the TEMPO…HF adduct, the hydrogen bond energy is 49 kJ/mol, while for the TEMPO…2HF adduct, it is 87 kJ/mol ≈ 2 × 44 kJ/mol. Therefore, similar to the P=O moiety, the N-O^•^ moiety in TEMPO can form two equally strong hydrogen bonds simultaneously.

This has important implications: in water and other protic solvents, adducts with two hydrogen bonds per TEMPO molecule are likely to exist. A_iso_(^14^N) in these adducts is larger than in 1:1 adducts. This may be another reason why the experimental A_iso_(^14^N) in water is greater than the calculated value for the 1:1 complex, as seen in Table 3. It also implies that the actual concentration of hydrogen-bonded TEMPO forms in alcohols is lower than what is estimated from the calculated A_iso_(^14^N) for 1:1 complexes.

Figure 4c shows the structure of the 1:1 complex of TEMPO with the pyridine-H^+^ cation. Both the geometry and A_iso_(^14^N) value were calculated using the PCM approximation in toluene. This cation is a stronger proton donor than alcohols and phenols. As a result, the hydrogen bond in this complex leads to an increase in the A_iso_(^14^N) value by more than 0.3 mT, as seen in Table 5. The presence of such complexes in solution should be easy to detect. The O…H distance in this complex is similar to the O…H distances in pyridine complexes with benzoic acid dimers [93]. However, the formation of the 1:2 adducts discussed earlier cannot be ruled out. In experimental studies of such systems, a balance must be struck between competing factors: the solvent must be sufficiently inert so as not to compete with the cation for interaction with TEMPO, while still maintaining sufficient solubility of the cation.

The *cis*-conformation of the 2,2′-bipyridine-H^+^ ([H-BiPy]^+^) cation is stabilized by a weak intramolecular hydrogen bond [94]. This bond plays a critical role in facilitating a reversible intramolecular proton transfer [95]. Figure 4d,e shows the structures of the 1:1 complexes of TEMPO with [H-BiPy]^+^ and [H-Phen]^+^. Both the geometry and A_iso_(^14^N) value were calculated using the PCM approximation in toluene. The significant differences in A_iso_(^14^N) and the O…H distances in these complexes are likely attributed to the effect of TEMPO on their intramolecular bonds, as seen in Table 5. In [H-Phen]^+^, this effect appears to be more pronounced compared to [H-BiPy]^+^. Nevertheless, the changes in A_iso_(^14^N) should be readily detectable experimentally for both complexes. It would be instructive to experimentally investigate whether a weak interaction with TEMPO can influence the energy balance in [H-BiPy]^+^ and alter its conformation from *cis* to *trans*.

### 2.6. Interaction of TEMPO with a Strong Halogen Bond Donor

**Figure 5:**
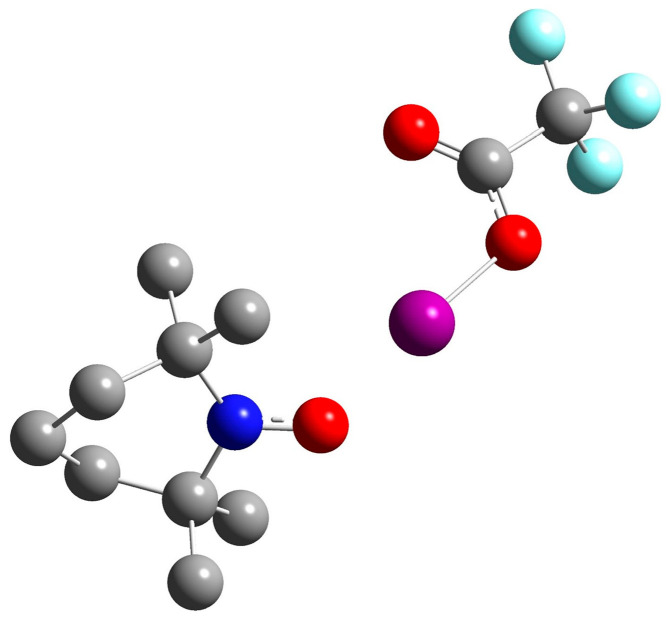
The model structure of the 1:1 complexes of TEMPO with iodo 2,2,2-trifluoroacetate in hexane. The calculated A_iso_(^14^N) value in this complex is 1.8635 mT. The calculated A_iso_(^14^N) value of TEMPO in hexane is 1.4996 mT. Oxygen atoms are represented in red, the nitrogen atom in blue, carbon atoms in grey, and the iodine atom in purple.

Figure 5 shows the structure of the 1:1 complex of TEMPO with iodo 2,2,2-trifluoroacetate. Both the geometry and A_iso_(^14^N) value were calculated using the PCM approximation in hexane. The choice of such a strong halogen bond donor [96] and a low polarity solvent was made deliberately to evaluate the maximum changes in A_iso_(^14^N) expected for halogen bonding. The calculated A_iso_(^14^N) value of TEMPO in hexane, equal to 1.4996 mT, increases to 1.8635 mT in this complex. The presence of such complexes in solution should be easily detected, even if their mole fraction is low.

## 3. Materials and Methods

The Gaussian 09.D.01 program package was used for geometry optimizations and EPR calculations [97], employing the ωB97XD/def2tzvp and uPBE1PBE/LanL2DZ DFT functional/basis set pairs, respectively [98,99,100,101]. The optimized geometries and the results of selected EPR calculations are reported in the Supplementary Materials. All calculations were performed using the PCM approximation. Although the results of such calculations are not highly sensitive to the specific value of the dielectric constant, applying this correction is important to account for the effects of the surrounding medium [102]. This study does not address whether other solvent models, such as the Solvation Model based on Density (SMD), the Conductor-like Polarizable Continuum Model (CPCM), or the Conductor-like Screening Model (COSMO), provide better approximations for medium effects. There is no reason to expect that TEMPO is a particularly convenient model system for answering this question. It is quite possible that when using one of these models, the uPBE1PBE/LanL2DZ DFT functional/basis set pair will no longer be the most suitable for EPR calculations. However, a more suitable pair can be easily found for the solvent model in question using the approach outlined in Table 1.

## 4. Conclusions

The isotropic hyperfine coupling constant A_iso_(^14^N) of the stable nitroxide radical TEMPO can be measured experimentally with an accuracy of 0.001 mT. The variation in this constant due solely to the polarity of the solvent is about 0.1 mT. The variations due to halogen and hydrogen bonding are greater than 0.3 mT and 0.5 mT, respectively. Therefore, A_iso_(^14^N) serves as a highly sensitive experimental probe for detecting the effects of the surrounding medium and non-covalent interactions.

A_iso_(^14^N) can be calculated for model systems of required complexity with reasonable accuracy using the uPBE1PBE/LanL2DZ approximation, which requires only moderate computational effort. If higher accuracy is desired, more sophisticated methods can be employed [103].

However, the most challenging aspect of such calculations is not the accuracy of the computational method but accounting for the effects of the surrounding medium and the presence of a variety of non-covalently bound complexes in solution, with diverse compositions and geometries. Each of these complexes exhibits a different A_iso_(^14^N) value. The structure and linewidth of the experimental spectrum depend on the relative concentration of these complexes, their lifetimes, and their tumbling times. If a study does not involve frozen solutions [104,105,106], interpreting the experimental spectra in terms of the structure and geometry of the potential complexes will require evaluating numerous model structures, the relative average concentration of which, in solution, cannot always be reliably predicted based on general assumptions [107]. Ideally, additional experimental measurements at very low concentrations should also be performed. In any case, for TEMPO, the list of possible model structures must include complexes featuring two conjugated hydrogen bonds.

This work examines the influence of various non-covalent interactions on A_iso_(^14^N) without a thorough analysis of the changes in the electron density distribution caused by these interactions, which, in turn, determines the observed changes in the A_iso_(^14^N) value. However, conducting such an analysis at a reliable level would require a separate, specialized study based on the implementation of appropriate experimental studies. Such studies are beyond the scope of this paper, although the results obtained here can help in planning the practical part of such studies.

## Figures and Tables

**Table 1:** A_iso_(^14^N) for TEMPO in toluene calculated for selected DFT functional/basis set pairs.

DFT Functional	Basis Set	A_iso_(^14^N), mT
―	―	1.551 ± 0.002 ^1^
uPBE1PBE	LanL2DZ	1.5147
uPBE1PBE	LanL2DZdp	1.4802
uPBE1PBE	EPR-III	1.0214
uPBE1PBE	6-31G-J	1.0222
uPBE1PBE	def2tzvp	0.9477
uPBE1PBE	aug-cc-pVqZ	0.8147
uHSEH1PBE	LanL2DZ	1.5154
uB3LYP	LanL2DZ	1.4629
uTPSSh	LanL2DZ	1.4233
uwB97XD	LanL2DZ	1.4559
uM06	LanL2DZ	1.1013

^1^ Experimental value [52].

**Table 2:** A_iso_(^14^N) for TEMPO in aprotic solvents.

Solvent	uPBE1PBE/LanL2DZ, mT	Experiment, mT
Hexane (ε ≈ 1.88)	1.4996	1.520 [70] 1.50 [74]
Cyclohexane (ε ≈ 2.02)	1.5042	1.540 [70] 1.51 [74]
CCl_4_ (ε ≈ 2.23)	1.5107	1.563 [75] 1.550 [70] 1.568 [35]
Benzene (ε ≈ 2.27)	―	1.554 [76]
Toluene (ε ≈ 2.37)	1.5147	1.551 [52]
Oxolane (THF, ε ≈ 7.43)	1.5671	1.557 [77]
Dichloromethane (ε ≈ 8.93)	―	1.587 [78]
DMSO (ε ≈ 46.83)	1.5959	―
Ionic Liquid	―	1.604 [46]
Liquid CO_2_	―	1.549 [79]
Supercritical CO_2_	―	1.545 [79]
Mineral oil	―	1.539 [46]

**Table 3:** A_iso_(^14^N) in 1:1 complexes of TEMPO and selected solvent molecules.

Hydrogen Bond Donor	uPBE1PBE/LanL2DZ, mT	Experiment, mT
2-methylpropan-2-ol ^1^	1.7108	1.60 [74]
Pentan-1-ol	―	1.61 [74]
3-methylbutan-1-ol	―	1.61 [74]
2-methylbutan-2-ol	―	1.58 [74]
Butan-2-ol	1.7256	1.60 [74]
Butan-1-ol	―	1.61 [74]
Propan-2-ol	―	1.61 [74]
Propan-1-ol	―	1.62 [74]
Ethanol	1.7337	1.632 [77] 1.62 [74]
Methanol ^1^	1.7371	1.63 [74]
Water ^1^	1.7274	1.769 [44] 1.732 [75]

^1^ A_iso_(^14^N) for TEMPO, calculated using the PCM approximation at ε = 78.36 (water), ε = 32.61 (methanol), and ε = 12.47 (2-methylpropan-2-ol), are 1.5984 mT, 1.5933 mT, and 1.5802 mT, respectively.

**Table 4:** A_iso_(^14^N) for TEMPO in 1:1 complexes with phenols in toluene. ^1^ The experimental values were obtained for TEMPO/phenol concentrations of 5 × 10^−4^ M/0.01 M and 5 × 10^−4^ M/0.05 M.

Phenol	uPBE1PBE/LanL2DZ, mT	Experiment [52], mT	
		0.01 M	0.05 M
Phenol	1.7415	1.565	1.607
4-nitrophenol	1.7852	1.595	1.633
2,5-dinitrophenol	1.8023	1.556	1.574
2,4,6-trinitrophenol	1.6242	1.552	1.557
2,6-di-*tert*-butylphenol	―	1.552	1.554

^1^ The experimental and calculated A_iso_(^14^N) values for TEMPO in toluene are 1.551 mT [52] and 1.5147 mT, respectively.

**Table 5:** The calculated A_iso_(^14^N) values of TEMPO in mT in complexes with selected proton donors.

Hydrogen-Bonded Complex	uPBE1PBE/LanL2DZ	O…H Distance, Å
TEMPO-H^+^ in water	2.4242 ^1^	0.97
TEMPO-2H^2+^ in water	2.4540 ^1^	0.99
TEMPO-HF in water	1.8508 ^1^	1.53
TEMPO-2HF in water	2.0509 ^1^	1.59
TEMPO-[H-pyridine]^+^ in toluene	1.8620 ^2^	1.61
TEMPO-[H-BiPy]^+^ in toluene	1.7468 ^2^	1.87
TEMPO-[H-Phen]^+^ in toluene	1.8145 ^2^	1.71

^1^ The calculated A_iso_(^14^N) value of TEMPO in water is 1.5984 mT. ^2^ The calculated A_iso_(^14^N) value of TEMPO in toluene is 1.5147 mT.

## Data Availability

Data are contained within the article and Supplementary Materials.

## Supplementary Materials

The following supporting information can be downloaded at: https://www.mdpi.com/article/10.3390/molecules29215032/s1, Table S1: The optimized molecular structure of TEMPO in toluene; Table S2: The optimized molecular structure of TEMPO in DMSO; Table S3: The optimized molecular structure of TEMPO in n-Hexane; Table S4: The optimized molecular structure of 1:1 complex of TEMPO and water; Table S5: The optimized molecular structure of 1:1 complex of TEMPO and methanol; Table S6: The optimized molecular structure of 1:1 complex of TEMPO and ethanol; Table S7: The optimized molecular structure of 1:1 complex of TEMPO and Butan-2-ol; Table S8: The optimized molecular structure of 1:1 complex of TEMPO and 2-methylpropan-2-ol; Table S9: The optimized molecular structure of 1:1 complex of TEMPO and phenol; Table S10: The optimized molecular structure of 1:1 complex of TEMPO and 4-nitrophenol; Table S11: The optimized molecular structure of 1:1 complex of TEMPO and 2,5-dinitrophenol; Table S12: The optimized molecular structure of 1:1 complex of TEMPO and 2,4,6-trinitrophenol; Table S13: The optimized molecular structure of [TEMPO-H]^+^; Table S14: The optimized molecular structure of [TEMPO-2H]^2+^; Table S15: The optimized molecular structure of 1:1 complex of TEMPO and HF; Table S16: The optimized molecular structure of 1:2 complex of TEMPO and HF; Table S17: The optimized molecular structure of 1:1 complex of TEMPO and [H-pyridine]^+^; Table S18: The optimized molecular structure of 1:1 complex of TEMPO and [H-BiPy]^+^; Table S19: The optimized molecular structure of 1:1 complex of TEMPO and [H-Phen]^+^; Table S20: The optimized molecular structure of 1:1 complex of TEMPO and iodo 2,2,2-trifluoroacetate; Table S21: The isotropic Fermi contact couplings in TEMPO in toluene; Table S22: The isotropic Fermi contact couplings in TEMPO in DMSO; Table S23: The isotropic Fermi contact couplings in TEMPO in n-hexane; and Table S24: The isotropic Fermi contact couplings in 1:1 complex of TEMPO and water.
