# Supplementary material for: Keep Your TEMPO Up: Nitroxide Radicals as Sensors of Intermolecular Interactions
Source: Molecules. 2024 Oct 24;29(21):5032. doi: 10.3390/molecules29215032 (PMC11548018; doi:10.3390/molecules29215032)
Supplement: Supplementary file 1 [file molecules-29-05032-s001.zip › molecules-3284779-supplementary.pdf]

molecules

Article

Keep Your TEMPO Up: Nitroxide Radicals as Sensors of Intermolecular Interactions

Ilya G. Shenderovich

Institute of Organic Chemistry, University of Regensburg, Universitaetstrasse 31, 93053 Regensburg, Germany
Ilya.Shenderovich@ur.de

Supplementary Materials:

Table S1. The optimized molecular structure of TEMPO calculated using the following approximation: wB97XD/def2tzvp opt=(VeryTight) int=ultrafine SCF=XQC SCRF=(Solvent=toluene) freq.

Atomic number	Coordinates, Å		
7	0.000000	0.794285	0.000001
8	-0.000001	2.062346	-0.000006
6	-1.302887	0.085963	0.001829
6	-1.110267	-1.295464	0.632634
1	-2.063703	-1.824426	0.581182
1	-0.904135	-1.146871	1.694593
6	0.000000	-2.141321	-0.000006
1	-0.416180	-2.797299	-0.766100
1	0.416180	-2.797305	0.766083
6	1.110269	-1.295460	-0.632640
1	0.904138	-1.146861	-1.694598
1	2.063705	-1.824422	-0.581190
6	1.302887	0.085964	-0.001826
6	2.288916	0.887169	-0.848291
1	2.482974	1.862754	-0.408398
1	3.229090	0.337274	-0.915562
1	1.900347	1.036537	-1.856470
6	1.826749	-0.009224	1.436192
1	2.831386	-0.436258	1.436604
1	1.874492	0.986661	1.877732
1	1.192389	-0.634486	2.064054
6	-1.826753	-0.009217	-1.436188
1	-2.831391	-0.436250	-1.436600
1	-1.874496	0.986671	-1.877723
1	-1.192396	-0.634476	-2.064055
6	-2.288913	0.887164	0.848301
1	-2.482971	1.862752	0.408414
1	-3.229088	0.337271	0.915572
1	-1.900340	1.036526	1.856480

Table S2. The optimized molecular structure of TEMPO calculated using the following approximation: wB97XD/def2tzvp opt=(VeryTight) int=ultrafine SCF=XQC SCRF=(Solvent= dimethylsulfoxide) freq.

Atomic number	Coordinates, Å		
7	0.000000	0.796355	0.000000
8	0.000000	2.066140	-0.000001
6	-1.303670	0.086783	0.001007
6	-1.110072	-1.294240	0.632468
1	-2.063676	-1.822030	0.578999
1	-0.905175	-1.145917	1.694602
6	0.000000	-2.139803	-0.000001
1	-0.416203	-2.794737	-0.766637
1	0.416203	-2.794737	0.766635
6	1.110072	-1.294240	-0.632468
1	0.905175	-1.145916	-1.694602
1	2.063676	-1.822030	-0.579000
6	1.303670	0.086783	-0.001007
6	2.293560	0.884064	-0.846759
1	2.504576	1.853935	-0.401383
1	3.226715	0.323676	-0.919570
1	1.904426	1.040772	-1.853759
6	1.824327	-0.009755	1.437965
1	2.824701	-0.445952	1.437504
1	1.882540	0.985818	1.879647
1	1.184425	-0.629072	2.065596
6	-1.824328	-0.009754	-1.437964
1	-2.824702	-0.445951	-1.437503
1	-1.882541	0.985820	-1.879646
1	-1.184426	-0.629070	-2.065596
6	-2.293559	0.884063	0.846760
1	-2.504575	1.853935	0.401385
1	-3.226715	0.323676	0.919571
1	-1.904425	1.040770	1.853761

Table S3. The optimized molecular structure of TEMPO calculated using the following approximation: wB97XD/def2tzvp opt=(VeryTight) int=ultrafine SCF=XQC SCRF=(Solvent= n-Hexane) freq.

Atomic number	Coordinates, Å		
7	0.000000	0.793771	0.000002
8	0.000000	2.061547	0.000002
6	-1.302771	0.085764	0.001755
6	-1.110468	-1.295762	0.632409
1	-2.063858	-1.824929	0.581120
1	-0.904344	-1.147115	1.694379
6	0.000000	-2.141644	-0.000006
1	-0.416001	-2.797795	-0.766108
1	0.416001	-2.797800	0.766092
6	1.110469	-1.295758	-0.632416
1	0.904344	-1.147105	-1.694385
1	2.063858	-1.824925	-0.581130
6	1.302771	0.085764	-0.001754
6	2.288193	0.887705	-0.848238
1	2.479134	1.864244	-0.409194
1	3.229625	0.339729	-0.914456
1	1.899871	1.035849	-1.856681
6	1.826996	-0.008911	1.436195
1	2.832372	-0.434319	1.437034
1	1.872847	0.987151	1.877487
1	1.193531	-0.635077	2.064147
6	-1.826997	-0.008903	-1.436194
1	-2.832373	-0.434311	-1.437035
1	-1.872848	0.987161	-1.877481
1	-1.193533	-0.635066	-2.064149
6	-2.288192	0.887700	0.848245
1	-2.479133	1.864242	0.409206
1	-3.229625	0.339724	0.914461
1	-1.899869	1.035838	1.856688

Table S4. The optimized molecular structure of 1:1 complex of TEMPO and water calculated using the following approximation: wB97XD/def2tzvp opt=(VeryTight) int=ultrafine SCF=XQC SCRF=(Solvent= water) freq.

Atomic number	Coordinates, Å		
7	-0.1792	0.35379	0.2605
8	-1.12832	1.11568	0.62876
6	-0.43989	-1.10177	0.12699
6	0.87707	-1.85235	0.34279
1	0.68178	-2.91457	0.18866
1	1.15068	-1.74396	1.39408
6	2.03401	-1.39092	-0.54907
1	2.08551	-2.00726	-1.44739
1	2.9699	-1.55392	-0.01329
6	1.91557	0.07663	-0.97235
1	1.41696	0.14557	-1.94111
1	2.90653	0.51171	-1.11002
6	1.14829	0.96751	0.00758
6	0.93598	2.33944	-0.62779
1	0.44394	3.02379	0.05993
1	1.9074	2.75536	-0.89794
1	0.33064	2.25819	-1.53161
6	1.8877	1.1281	1.34085
1	2.8252	1.66075	1.1741
1	1.28028	1.70833	2.0364
1	2.11967	0.16848	1.80117
6	-1.03814	-1.37992	-1.25705
1	-1.30924	-2.43435	-1.329
1	-1.93835	-0.78199	-1.4053
1	-0.33707	-1.15397	-2.05955
6	-1.43096	-1.52373	1.20905
1	-2.40805	-1.0693	1.06047
1	-1.54847	-2.60734	1.17305
1	-1.06668	-1.24694	2.19899
8	-3.69641	1.11325	-0.46549
1	-2.81606	1.0483	-0.05471
1	-4.18008	0.34722	-0.15266

Table S5. The optimized molecular structure of 1:1 complex of TEMPO and methanol calculated using the following approximation: wB97XD/def2tzvp opt=(VeryTight) int=ultrafine SCF=XQC SCRF=(Solvent= methanol) freq.

Atomic number	Coordinates, Å		
7	0.05464	0.08713	0.50659
8	-0.93079	0.32845	1.27364
6	0.32916	-1.31689	0.10498
6	1.83865	-1.46685	-0.103
1	2.02912	-2.4884	-0.43535
1	2.3174	-1.37074	0.87351
6	2.4512	-0.46548	-1.08773
1	2.49311	-0.89916	-2.0876
1	3.48444	-0.27765	-0.79327
6	1.67456	0.85317	-1.16237
1	0.94813	0.8128	-1.9762
1	2.34949	1.67645	-1.40097
6	0.92111	1.22672	0.11585
6	0.03119	2.43584	-0.16291
1	-0.48675	2.76189	0.73658
1	0.65243	3.25571	-0.52543
1	-0.71195	2.20057	-0.92605
6	1.87644	1.54528	1.27166
1	2.45301	2.43946	1.03001
1	1.30904	1.73614	2.18305
1	2.57517	0.73133	1.46213
6	-0.46031	-1.64781	-1.16739
1	-0.28912	-2.69176	-1.43503
1	-1.52805	-1.50943	-0.9936
1	-0.16175	-1.02842	-2.01197
6	-0.11649	-2.2447	1.23155
1	-1.19462	-2.2092	1.37491
1	0.16415	-3.26643	0.97302
1	0.3689	-1.9786	2.17112
8	-3.4244	-0.22176	0.20296
1	-2.57864	-0.11812	0.67097
6	-3.43937	0.72115	-0.84646
1	-4.37965	0.60831	-1.38658
1	-3.37516	1.7493	-0.47392
1	-2.61656	0.55995	-1.55334

Table S6. The optimized molecular structure of 1:1 complex of TEMPO and ethanol calculated using the following approximation: wB97XD/def2tzvp opt=(VeryTight) int=ultrafine SCF=XQC SCRF=(Solvent= ethanol) freq.

Atomic number	Coordinates, Å		
7	0.48117	-0.05994	-0.5701
8	-0.38349	-0.22411	-1.48823
6	0.76264	1.31461	-0.0804
6	2.23047	1.37452	0.35114
1	2.42218	2.37438	0.74327
1	2.84277	1.27747	-0.54763
6	2.63761	0.31424	1.37945
1	2.54806	0.71648	2.38931
1	3.69334	0.08044	1.23679
6	1.7958	-0.96299	1.28863
1	0.95991	-0.90941	1.98875
1	2.38752	-1.82822	1.59112
6	1.2226	-1.25598	-0.09939
6	0.24213	-2.42246	-0.00017
1	-0.15	-2.69366	-0.97808
1	0.76016	-3.28512	0.42068
1	-0.59488	-2.16887	0.65178
6	2.32138	-1.59251	-1.11399
1	2.8073	-2.52611	-0.82648
1	1.88652	-1.72068	-2.10568
1	3.08309	-0.81539	-1.1672
6	-0.18807	1.65154	1.07481
1	-0.00149	2.6743	1.40651
1	-1.22408	1.58311	0.74114
1	-0.05239	0.98882	1.92837
6	0.53541	2.29842	-1.22444
1	-0.51006	2.32616	-1.52515
1	0.82507	3.29514	-0.88935
1	1.14064	2.03438	-2.09228
8	-2.97359	0.45045	-0.76297
1	-2.08132	0.30268	-1.11945
6	-3.19399	-0.5218	0.24152
1	-3.16227	-1.53029	-0.18912
1	-2.40395	-0.46978	1.00221
6	-4.54014	-0.2742	0.88319
1	-5.33706	-0.33498	0.13975
1	-4.73453	-1.0183	1.65723
1	-4.57112	0.71595	1.34165

Table S7. The optimized molecular structure of 1:1 complex of TEMPO and Butan-2-ol calculated using the following approximation: wB97XD/def2tzvp opt=(VeryTight) int=ultrafine SCF=XQC SCRF=(Solvent= 2-Butanol) freq.

Atomic number	Coordinates, Å		
7	0.98199	0.18145	0.56368
8	0.21524	0.60467	1.48585
6	1.18536	-1.28283	0.40486
6	2.61134	-1.51157	-0.10389
1	2.74486	-2.58435	-0.25107
1	3.2975	-1.22648	0.69614
6	2.95902	-0.75245	-1.38861
1	2.77643	-1.38374	-2.25911
1	4.02846	-0.53806	-1.38653
6	2.15971	0.54461	-1.55227
1	1.26594	0.35932	-2.15114
1	2.74464	1.28252	-2.10329
6	1.71435	1.19155	-0.23952
6	0.76339	2.34587	-0.54674
1	0.45956	2.8595	0.36287
1	1.27007	3.0585	-1.19875
1	-0.13137	1.98584	-1.05652
6	2.90421	1.7107	0.57595
1	3.3907	2.52207	0.0324
1	2.55841	2.09639	1.53537
1	3.64502	0.93363	0.76116
6	0.13662	-1.84679	-0.56137
1	0.27315	-2.92612	-0.64841
1	-0.86688	-1.65754	-0.17997
1	0.21729	-1.41483	-1.55801
6	1.02504	-1.95047	1.76741
1	0.00671	-1.85807	2.1396
1	1.26004	-3.01067	1.66508
1	1.70474	-1.51315	2.49943
8	-2.42715	-0.20879	1.16231
1	-1.52353	0.03045	1.42756
6	-2.72597	0.52474	-0.0161
6	-2.90649	2.00155	0.2979
1	-2.01222	2.39612	0.78316
1	-3.08116	2.57517	-0.61504
1	-3.75778	2.14474	0.96758
6	-3.96412	-0.08667	-0.65167
1	-4.78258	-0.0337	0.07356
1	-4.25522	0.53673	-1.5013
1	-1.89059	0.4171	-0.72424
6	-3.76214	-1.52383	-1.11116
1	-3.48781	-2.1655	-0.27317
1	-4.67146	-1.92415	-1.56246
1	-2.96347	-1.58526	-1.85477

Table S8. The optimized molecular structure of 1:1 complex of TEMPO and 2-methylpropan-2-ol calculated using the following approximation: wB97XD/def2tzvp opt=(VeryTight) int=ultrafine SCF=XQC SCRF=(Solvent= 2-Methyl-2-Propanol) freq.

Atomic number	Coordinates, Å		
7	-0.92412	0.08362	-0.48638
8	0.09993	0.28321	-1.21224
6	-1.31987	-1.31394	-0.17377
6	-2.83276	-1.3477	0.05808
1	-3.10252	-2.36683	0.33894
1	-3.31927	-1.1594	-0.90089
6	-3.34314	-0.35601	1.10831
1	-3.39381	-0.83683	2.08601
1	-4.36608	-0.07694	0.85261
6	-2.46688	0.89559	1.22624
1	-1.73109	0.76126	2.02181
1	-3.07224	1.75602	1.51532
6	-1.70651	1.26623	-0.04889
6	-0.73731	2.40515	0.25797
1	-0.20034	2.71969	-0.63437
1	-1.30307	3.25415	0.64363
1	-0.00921	2.10585	1.0128
6	-2.64892	1.69314	-1.18053
1	-3.15772	2.61683	-0.90045
1	-2.07747	1.87665	-2.09102
1	-3.40635	0.93994	-1.39318
6	-0.54256	-1.79782	1.05539
1	-0.80524	-2.83668	1.26197
1	0.52958	-1.74526	0.86629
1	-0.7665	-1.20713	1.94314
6	-0.97858	-2.19861	-1.37033
1	0.09595	-2.25748	-1.5314
1	-1.35365	-3.20483	-1.17897
1	-1.44736	-1.82075	-2.27966
8	2.59125	-0.89939	-0.67809
1	1.7101	-0.54168	-0.88085
6	3.2832	0.05339	0.12745
6	3.30209	1.40779	-0.57886
1	2.28284	1.77443	-0.71913
1	3.85614	2.14461	0.0062
1	3.7752	1.31568	-1.55831
6	2.59475	0.17625	1.48617
1	2.58408	-0.78902	1.99572
1	3.10941	0.89918	2.12228
1	1.56201	0.50708	1.35819
6	4.69607	-0.48616	0.29379
1	4.67311	-1.46817	0.76987
1	5.18166	-0.58433	-0.67876
1	5.29367	0.18431	0.91356

Table S9. The optimized molecular structure of 1:1 complex of TEMPO and phenol calculated using the following approximation: wB97XD/def2tzvp opt=(VeryTight) int=ultrafine SCF=XQC SCRF=(Solvent =toluene) freq.

Atomic number	Coordinates, Å		
7	1.39346	-0.05605	-0.52141
8	0.50602	-0.42919	-1.35094
6	1.30977	1.31805	0.03247
6	2.71783	1.76535	0.43259
1	2.63628	2.75479	0.88534
1	3.29544	1.89936	-0.48427
6	3.45127	0.80842	1.37832
1	3.29767	1.11274	2.41447
1	4.52313	0.89186	1.19354
6	2.99936	-0.64796	1.2268
1	2.21304	-0.87137	1.95029
1	3.8232	-1.32544	1.45718
6	2.4677	-1.01453	-0.16071
6	1.87216	-2.41922	-0.11423
1	1.52193	-2.73359	-1.09511
1	2.64116	-3.11625	0.22127
1	1.03505	-2.46556	0.58327
6	3.56262	-0.95675	-1.23248
1	4.3118	-1.72385	-1.02983
1	3.1304	-1.14756	-2.21517
1	4.0648	0.00984	-1.25716
6	0.34565	1.31815	1.22467
1	0.22847	2.338	1.59497
1	-0.63455	0.95225	0.91815
1	0.7041	0.69721	2.04475
6	0.7769	2.24971	-1.0528
1	-0.24925	2.00402	-1.31583
1	0.80343	3.27452	-0.67991
1	1.38988	2.19051	-1.95284
6	-4.77122	-0.32496	0.94146
6	-4.90561	0.74868	0.07082
6	-3.89237	1.0098	-0.83939
6	-2.75771	0.21438	-0.8847
6	-2.62575	-0.85661	-0.0032
6	-3.64142	-1.12484	0.91045
1	-5.55521	-0.54287	1.65648
1	-5.79007	1.37135	0.10067
1	-3.98351	1.84046	-1.52873
1	-1.96829	0.41307	-1.59905
1	-3.52854	-1.96086	1.58887
8	-1.5399	-1.6586	0.00124
1	-0.84856	-1.29399	-0.58415

Table S10. The optimized molecular structure of 1:1 complex of TEMPO and 4-nitrophenol calculated using the following approximation: wB97XD/def2tzvp opt=(VeryTight) int=ultrafine SCF=XQC SCRF=(Solvent =toluene) freq.

Atomic number	Coordinates, Å		
7	2.30426	0.02152	-0.49453
8	1.43839	-0.41419	-1.31745
6	2.10263	1.3729	0.08443
6	3.46309	1.93068	0.5094
1	3.29043	2.89525	0.98913
1	4.03148	2.14332	-0.39808
6	4.27456	1.01668	1.43338
1	4.08908	1.27556	2.47653
1	5.33521	1.20203	1.2591
6	3.95745	-0.46943	1.23691
1	3.19129	-0.78425	1.94828
1	4.8382	-1.07603	1.45325
6	3.4662	-0.84155	-0.16407
6	3.01144	-2.29829	-0.16542
1	2.68462	-2.61167	-1.15471
1	3.84935	-2.92655	0.13875
1	2.19195	-2.45424	0.5371
6	4.54914	-0.6431	-1.23118
1	5.3705	-1.33767	-1.04842
1	4.1381	-0.84771	-2.22015
1	4.953	0.36854	-1.22694
6	1.1316	1.26209	1.26573
1	0.91404	2.25923	1.6515
1	0.19321	0.81057	0.94191
1	1.53987	0.66368	2.07933
6	1.5038	2.27974	-0.98776
1	0.50191	1.96085	-1.26523
1	1.44637	3.2961	-0.59627
1	2.12557	2.28662	-1.88355
6	-3.83413	-1.19485	0.75977
6	-4.04398	-0.04615	0.0092
6	-3.0498	0.47316	-0.80515
6	-1.82722	-0.16115	-0.86861
6	-1.59618	-1.31342	-0.11094
6	-2.61215	-1.82664	0.69928
1	-4.6274	-1.57934	1.3838
1	-3.24149	1.36466	-1.38408
1	-1.03918	0.22257	-1.50303
1	-2.42084	-2.72104	1.27643
8	-0.4273	-1.95863	-0.12934
1	0.24061	-1.46161	-0.64969
7	-5.33342	0.62594	0.07819
8	-5.48852	1.63844	-0.57796
8	-6.19387	0.14372	0.7897

Table S11. The optimized molecular structure of 1:1 complex of TEMPO and 2,5-dinitrophenol calculated using the following approximation: wB97XD/def2tzvp opt=(VeryTight) int=ultrafine SCRF=(Solvent=toluene) freq.

Atomic number	Coordinates, Å		
7	2.28144	-0.22173	-0.40248
8	1.27431	-0.34158	-1.16976
6	2.71051	1.1509	-0.02998
6	4.21632	1.13132	0.24363
1	4.50554	2.13007	0.57377
1	4.7232	0.97053	-0.70981
6	4.67149	0.08307	1.26408
1	4.71966	0.52381	2.26054
1	5.68962	-0.22082	1.01779
6	3.75248	-1.14183	1.31766
1	3.00421	-1.01398	2.10243
1	4.32354	-2.03107	1.58838
6	3.01009	-1.44562	0.01438
6	1.9999	-2.56229	0.26279
1	1.51184	-2.8764	-0.65761
1	2.52514	-3.42277	0.67831
1	1.23552	-2.25096	0.97574
6	3.96387	-1.86226	-1.1115
1	4.42767	-2.81665	-0.85766
1	3.40958	-1.98536	-2.0422
1	4.75597	-1.13238	-1.27322
6	1.91113	1.60148	1.19855
1	2.16092	2.63735	1.43209
1	0.84165	1.55208	0.99267
1	2.1248	0.99278	2.07637
6	2.42522	2.09066	-1.19849
1	1.35812	2.20429	-1.37406
1	2.83802	3.07246	-0.96375
1	2.89271	1.72619	-2.11363
6	-4.10549	-0.10858	0.50943
6	-3.81043	1.23742	0.45428
6	-2.56051	1.59556	-0.0208
6	-1.6188	0.68282	-0.43673
6	-1.91285	-0.68185	-0.39111
6	-3.18	-1.04418	0.07993
1	-5.05979	-0.45025	0.88407
1	-4.51866	1.98654	0.77053
1	-0.65444	1.00588	-0.80085
8	-1.03321	-1.60863	-0.74065
1	-0.15114	-1.20615	-0.91992
7	-3.57177	-2.45395	0.14246
8	-3.29778	-3.16044	-0.79976
8	-4.17358	-2.81574	1.134
7	-2.20812	3.02748	-0.07924
8	-1.0784	3.31956	-0.40944
8	-3.06786	3.82965	0.21037

Table S12. The optimized molecular structure of 1:1 complex of TEMPO and 2,4,6-trinitrophenol calculated using the following approximation: wB97XD/def2tzvp opt=(VeryTight) int=ultrafine SCRF=(Solvent=toluene) freq.

Atomic number	Coordinates, Å		
7	2.13274	-0.41469	0.23356
8	1.40858	-0.2469	1.26046
6	1.82059	-1.54044	-0.67972
6	3.11182	-1.97256	-1.37819
1	2.86358	-2.76951	-2.08125
1	3.75907	-2.42482	-0.62428
6	3.85565	-0.84209	-2.09784
1	3.55689	-0.80334	-3.14636
1	4.92187	-1.07254	-2.09562
6	3.60986	0.53434	-1.46906
1	2.78766	1.03832	-1.98108
1	4.48451	1.1726	-1.60424
6	3.27663	0.50596	0.02415
6	2.85882	1.90381	0.47276
1	2.67342	1.93158	1.54453
1	3.66147	2.60494	0.23919
1	1.95319	2.22561	-0.04179
6	4.46383	0.03017	0.86961
1	5.27485	0.75747	0.80348
1	4.16275	-0.05701	1.91396
1	4.84607	-0.93498	0.53844
6	0.75425	-1.09042	-1.68441
1	0.45732	-1.93217	-2.31228
1	-0.12573	-0.73686	-1.14843
1	1.10642	-0.28797	-2.33134
6	1.27873	-2.70286	0.14739
1	0.33475	-2.44545	0.62392
1	1.11675	-3.55995	-0.50774
1	1.98518	-2.98807	0.92729
6	-2.63451	0.55715	-1.01187
6	-2.85792	-0.64825	-0.37207
6	-2.29577	-0.92517	0.85405
6	-1.5044	0.02847	1.45671
6	-1.22777	1.27154	0.84898
6	-1.81111	1.47747	-0.41349
1	-3.08447	0.76629	-1.97149
1	-2.47167	-1.87126	1.34355
8	-0.51128	2.21837	1.39519
1	-0.20825	1.89151	2.26921
7	-1.53653	2.71513	-1.152
8	-0.3898	3.09448	-1.19942
8	-2.479	3.25026	-1.69326
7	-3.69992	-1.65608	-1.01804
8	-3.87435	-2.70079	-0.43021
8	-4.16682	-1.38191	-2.10219
7	-0.9483	-0.28988	2.76667
8	-0.41364	0.61686	3.3936
8	-1.05918	-1.41393	3.17945

Table S13. The optimized molecular structure of $[TEMPO\text{-}H]^+$ calculated using the following approximation: wB97XD/def2tzvp opt=(VeryTight) int=ultrafine SCF=XQC SCRF=(Solvent=water) freq.

Atomic number	Coordinates, Å		
7	-0.00144	-0.72605	-0.00062
8	0.05641	-2.03661	-0.04294
6	1.32356	-0.06786	0.01375
6	1.12918	1.33111	0.60066
1	2.088	1.84151	0.51643
1	0.93739	1.22344	1.66874
6	0.02483	2.16375	-0.05989
1	0.44421	2.77941	-0.85476
1	-0.37394	2.85197	0.68444
6	-1.1074	1.32002	-0.65656
1	-0.93184	1.14376	-1.71855
1	-2.05483	1.85352	-0.588
6	-1.31849	-0.04086	0.01278
6	-2.326	-0.86288	-0.79008
1	-2.63111	-1.78229	-0.28342
1	-3.23036	-0.26577	-0.89039
1	-1.95862	-1.09022	-1.79082
6	-1.77729	0.08162	1.47322
1	-2.77298	0.5243	1.47272
1	-1.83487	-0.89758	1.94828
1	-1.11926	0.71799	2.05968
6	1.8347	-0.02817	-1.43424
1	2.83298	0.40828	-1.42386
1	1.89905	-1.03417	-1.84703
1	1.20066	0.57899	-2.07746
6	2.27407	-0.88017	0.89238
1	2.4998	-1.85077	0.45625
1	3.20343	-0.31906	0.97999
1	1.86038	-1.02224	1.89064
1	-0.84232	-2.38522	-0.16714

Table S14. The optimized molecular structure of $[TEMPO\text{-}2H]^{2+}$ calculated using the following approximation: wB97XD/def2tzvp opt=(VeryTight) int=ultrafine SCF=XQC SCRF=(Solvent=water) freq.

Atomic number	Coordinates, Å		
7	-0.00557	-0.64567	-0.03817
8	-0.05025	-2.04566	0.11163
6	1.32333	-0.02829	-0.01674
6	1.1293	1.33477	0.67222
1	2.08735	1.84465	0.58474
1	0.97295	1.14331	1.73329
6	0.00254	2.20025	0.10077
1	0.40172	2.8971	-0.63343
1	-0.40297	2.8019	0.91205
6	-1.1193	1.39768	-0.56956
1	-0.95313	1.32453	-1.64396
1	-2.07659	1.89994	-0.4417
6	-1.32107	-0.02155	-0.02998
6	-2.29934	-0.81724	-0.90146
1	-2.56578	-1.77593	-0.45781
1	-3.21243	-0.22868	-0.96121
1	-1.91885	-0.95016	-1.91452
6	-1.80419	-0.02786	1.44993
1	-2.7922	0.43012	1.43243
1	-1.88705	-1.04352	1.82962
1	-1.1515	0.55518	2.0932
6	1.77406	0.13054	-1.49534
1	2.757	0.59843	-1.4498
1	1.86253	-0.83228	-1.99432
1	1.1045	0.77291	-2.05891
6	2.31199	-0.89924	0.75535
1	2.59403	-1.81208	0.22236
1	3.23369	-0.32837	0.84445
1	1.97037	-1.11835	1.76798
1	-0.39177	-2.50857	-0.68963
1	0.82501	-2.39969	0.40818

Table S15. The optimized molecular structure of 1:1 complex of TEMPO and HF calculated using the following approximation: wB97XD/def2tzvp opt=(VeryTight) int=ultrafine SCF=XQC SCRF=(Solvent=water) freq.

Atomic number	Coordinates, Å		
7	-0.1888	0.39305	0.17098
8	-1.10372	1.25048	0.40142
6	-0.54465	-1.04738	0.10051
6	0.70404	-1.87084	0.42835
1	0.44226	-2.92251	0.3054
1	0.9205	-1.73601	1.48949
6	1.94186	-1.53196	-0.40676
1	1.99711	-2.18392	-1.27913
1	2.82915	-1.74489	0.19039
6	1.96171	-0.07675	-0.88452
1	1.53769	-0.00788	-1.88823
1	2.98865	0.28232	-0.96339
6	1.19609	0.90311	0.00815
6	1.13642	2.26398	-0.68131
1	0.65665	3.01	-0.05159
1	2.15514	2.59057	-0.89273
1	0.59119	2.19889	-1.62357
6	1.84055	1.05604	1.39067
1	2.82426	1.51402	1.27882
1	1.22823	1.70355	2.01911
1	1.96685	0.10015	1.89659
6	-1.08214	-1.35701	-1.30127
1	-1.41415	-2.39548	-1.33646
1	-1.934	-0.71694	-1.53212
1	-0.32308	-1.21557	-2.06975
6	-1.61507	-1.34606	1.14943
1	-2.56529	-0.87236	0.91328
1	-1.77451	-2.42425	1.18232
1	-1.29195	-1.01503	2.137
1	-2.56204	1.1033	-0.03861
9	-3.48379	1.11311	-0.3294

Table S16. The optimized molecular structure of 1:2 complex of TEMPO and HF calculated using the following approximation: wB97XD/def2tzvp opt=(VeryTight) int=ultrafine SCF=XQC SCRF=(Solvent=water) freq.

Atomic number	Coordinates, Å		
7	-0.130178	-0.000001	0.000001
8	-1.410347	-0.000005	0.000005
6	0.568552	-1.279187	0.288792
6	1.948666	-0.943459	0.860598
1	2.473283	-1.886911	1.014224
1	1.800058	-0.514601	1.853101
6	2.793456	0.000010	0.000002
1	3.447216	-0.573745	-0.657300
1	3.447206	0.573772	0.657306
6	1.948659	0.943469	-0.860599
1	1.800055	0.514607	-1.853100
1	2.473270	1.886924	-1.014228
6	0.568543	1.279188	-0.288793
6	-0.228456	2.049129	-1.340663
1	-1.178386	2.411068	-0.953672
1	0.360469	2.912570	-1.650181
1	-0.417625	1.428202	-2.216643
6	0.662250	2.098388	1.003654
1	1.107787	3.066761	0.773673
1	-0.329707	2.269083	1.422061
1	1.278447	1.610081	1.756789
6	0.662264	-2.098384	-1.003656
1	1.107802	-3.066756	-0.773676
1	-0.329692	-2.269081	-1.422066
1	1.278462	-1.610075	-1.756790
6	-0.228442	-2.049134	1.340661
1	-1.178369	-2.411081	0.953668
1	0.360489	-2.912571	1.650178
1	-0.417617	-1.428209	2.216641
1	-2.316566	-1.197408	-0.531405
1	-2.316572	1.197398	0.531409
9	-2.912714	-1.858367	-0.874867
9	-2.912724	1.858356	0.874867

Table S17. The optimized molecular structure of 1:1 complex of TEMPO and [H-pyridine]+ calculated using the following approximation: wB97XD/def2tzvp opt=(VeryTight) int=ultrafine SCF=XQC SCRF=(Solvent =toluene) freq.

Atomic number	Coordinates, Å		
7	-1.14487	0.0513	-0.3684
8	-0.04534	0.19581	-0.99283
6	-1.58296	-1.31832	0.00246
6	-3.11325	-1.33788	0.05586
1	-3.41925	-2.32956	0.39153
1	-3.47928	-1.24073	-0.96776
6	-3.74401	-0.25948	0.94282
1	-3.93536	-0.65552	1.94042
1	-4.71837	0.00067	0.52886
6	-2.87461	0.99457	1.07659
1	-2.24961	0.92195	1.96898
1	-3.50158	1.87543	1.21977
6	-1.9549	1.26914	-0.1149
6	-1.00455	2.41194	0.23547
1	-0.37192	2.67462	-0.61019
1	-1.59217	3.28713	0.51293
1	-0.37388	2.14325	1.08498
6	-2.73653	1.61823	-1.3861
1	-3.26027	2.56365	-1.23962
1	-2.05261	1.7292	-2.2277
1	-3.47485	0.85817	-1.63735
6	-0.95458	-1.68799	1.35089
1	-1.1986	-2.72302	1.59291
1	0.13219	-1.59973	1.30094
1	-1.31422	-1.05667	2.16196
6	-1.11001	-2.29384	-1.07178
1	-0.02566	-2.38286	-1.08903
1	-1.52924	-3.27733	-0.85863
1	-1.44583	-1.98008	-2.06004
6	4.26482	1.30738	0.4134
6	5.0923	0.19365	0.40146
6	4.58257	-1.04483	0.03747
6	3.25399	-1.14049	-0.30488
6	2.94401	1.15399	0.0618
1	4.63657	2.28287	0.68937
1	6.13462	0.29034	0.67417
1	5.20532	-1.92666	0.01734
1	2.77998	-2.06449	-0.6019
1	2.22774	1.96289	0.03799
1	1.46675	-0.11584	-0.54205
7	2.48038	-0.0514	-0.28139

Table S18. The optimized molecular structure of 1:1 complex of TEMPO and [H-BiPy]$^+$ calculated using the following approximation: wB97XD/def2tzvp opt=(VeryTight) int=ultrafine SCF=XQC SCRF=(Solvent=toluene) freq.

Atomic number	Coordinates, Å		
7	-2.0752	-0.06114	-0.24343
8	-0.92804	0.28409	-0.66607
6	-2.16476	-1.10953	0.80266
6	-3.54033	-1.77492	0.71127
1	-3.6136	-2.49618	1.52648
1	-3.56254	-2.35943	-0.21028
6	-4.73081	-0.81171	0.74713
1	-5.10003	-0.70558	1.76772
1	-5.54786	-1.25119	0.17424
6	-4.39295	0.58044	0.20467
1	-4.10906	1.24082	1.02676
1	-5.27414	1.03199	-0.25327
6	-3.2628	0.61241	-0.82733
6	-2.90117	2.06562	-1.12434
1	-2.11653	2.13495	-1.8748
1	-3.78674	2.57987	-1.49854
1	-2.57277	2.5768	-0.21702
6	-3.64654	-0.09607	-2.1312
1	-4.46076	0.44577	-2.61436
1	-2.79409	-0.11486	-2.8108
1	-3.97589	-1.12017	-1.96142
6	-1.92804	-0.45898	2.17042
1	-1.90136	-1.22874	2.94283
1	-0.9697	0.06295	2.17632
1	-2.71178	0.25263	2.42818
6	-1.08716	-2.15752	0.53855
1	-0.08817	-1.74253	0.65309
1	-1.21176	-2.97558	1.24911
1	-1.17864	-2.55903	-0.47168
7	1.51605	1.22287	0.00535
6	3.1285	3.31719	0.5432
7	2.255	-1.28392	-0.23839
6	2.83285	0.98454	0.09868
6	2.54128	-2.56079	-0.43225
6	0.96541	2.42925	0.15662
6	3.25788	-0.41849	-0.10705
6	4.59124	-0.79486	-0.16742
6	3.6674	2.05311	0.37664
6	1.75929	3.5158	0.43126
6	4.883	-2.13445	-0.37053
6	3.84396	-3.03614	-0.50536
1	1.69674	-3.23296	-0.53192
1	5.39105	-0.07552	-0.06797
1	5.9117	-2.46452	-0.42387
1	4.03097	-4.08875	-0.66503
1	-0.10774	2.46274	0.03875
1	1.3156	4.4923	0.55258
1	3.78094	4.15195	0.76176
1	4.73168	1.90139	0.46799
1	0.88037	0.43409	-0.19464

Table S19. The optimized molecular structure of 1:1 complex of TEMPO and [H-Phen]+ calculated using the following approximation: wB97XD/def2tzvp opt=(VeryTight) int=ultrafine SCF=XQC SCRF=(Solvent=toluene) freq.

Atomic number	Coordinates, Å		
7	2.24899	-0.55585	0.19901
8	1.2746	-1.26787	0.59413
6	2.60868	-0.5756	-1.24164
6	4.10546	-0.28099	-1.36622
1	4.35084	-0.24617	-2.42857
1	4.64769	-1.13623	-0.95882
6	4.561	1.0087	-0.67503
1	4.55739	1.83705	-1.38433
1	5.59752	0.885	-0.36011
6	3.6864	1.39008	0.52463
1	2.90476	2.08592	0.21158
1	4.27968	1.91944	1.27137
6	3.00469	0.21039	1.22026
6	2.0145	0.73436	2.25722
1	1.57308	-0.07935	2.82881
1	2.54139	1.39644	2.94509
1	1.20997	1.29147	1.77657
6	4.0151	-0.72145	1.89896
1	4.49955	-0.19162	2.72031
1	3.50331	-1.59352	2.30663
1	4.78909	-1.06222	1.21269
6	1.7606	0.46006	-1.98826
1	1.9409	0.3687	-3.06032
1	0.70073	0.2929	-1.7966
1	2.00134	1.4801	-1.69115
6	2.3184	-1.9681	-1.79525
1	1.25136	-2.18409	-1.79354
1	2.67754	-2.01935	-2.82347
1	2.82872	-2.73444	-1.21129
1	-0.39193	-1.01197	0.29713
7	-1.35253	-1.40972	0.24044
6	-1.82316	3.50185	-0.15329
6	-3.10802	3.03431	-0.21015
6	-3.33842	1.65012	-0.1243
6	-2.21167	0.82732	0.01718
6	-0.77127	2.58321	-0.01076
6	-4.6472	1.0711	-0.17758
6	-2.4161	-0.59039	0.10286
6	-3.70159	-1.14077	0.04453
6	-4.82444	-0.26418	-0.0981
6	-3.82195	-2.53696	0.13057
1	-4.80697	-2.98457	0.08792
6	-2.70712	-3.33036	0.26743
6	-1.46036	-2.72622	0.32168
1	-5.49775	1.73236	-0.28437
1	-1.608	4.55937	-0.21612
1	-3.94583	3.71112	-0.3213
1	0.25452	2.93366	0.03467
1	-5.81492	-0.6974	-0.14046
1	-2.78211	-4.4051	0.3347
1	-0.53242	-3.2691	0.43191
7	-0.95182	1.28638	0.07335

Table S20. The optimized molecular structure of 1:1 complex of TEMPO and iodo 2,2,2-trifluoroacetate calculated using the following approximation: wB97XD/def2tzvp opt=(VeryTight) int=ultrafine SCF=XQC SCRF=(Solvent= n-Hexane) freq.

Atomic number	Coordinates, Å		
7	2.54124	-0.14566	-0.23471
8	1.67291	-0.73059	-0.95718
6	2.76166	1.3186	-0.38663
6	4.26471	1.57287	-0.23062
1	4.42864	2.64784	-0.31798
1	4.76674	1.11835	-1.08711
6	4.87036	1.04724	1.07528
1	4.88558	1.8368	1.82734
1	5.91238	0.78228	0.89256
6	4.11071	-0.15413	1.64824
1	3.36532	0.18281	2.37032
1	4.79099	-0.80288	2.20173
6	3.3996	-1.01896	0.60703
6	2.49846	-2.03069	1.31228
1	2.01427	-2.69346	0.59805
1	3.10503	-2.63146	1.99073
1	1.72735	-1.52458	1.89488
6	4.39063	-1.74993	-0.30549
1	4.96182	-2.4659	0.2868
1	3.85321	-2.29345	-1.08214
1	5.09228	-1.06512	-0.78093
6	1.94232	2.07798	0.66469
1	2.07874	3.14906	0.50844
1	0.88067	1.85327	0.56584
1	2.24985	1.84395	1.68217
6	2.31909	1.7396	-1.78421
1	1.24414	1.6348	-1.91679
1	2.57997	2.78892	-1.92529
1	2.82187	1.1494	-2.55023
8	-2.78389	-0.55425	-0.35051
6	-3.30281	0.40892	0.353
8	-2.75595	1.32608	0.8972
6	-4.84773	0.23143	0.42464
9	-5.4035	1.20835	1.12756
9	-5.16272	-0.92939	1.00497
9	-5.38353	0.24217	-0.79812
53	-0.72653	-0.56922	-0.6134

Table S21. The isotropic Fermi contact couplings in TEMPO calculated using the following approximation: uPBE1PBE/LanL2DZ prop=EPR nosymm int=ultrafine iop(3/75=7) SCRF=(Solvent=toluene).

Atom	Gauss
N(14)	15.14687
O(17)	-28.17555
C(13)	-7.0556
C(13)	2.64134
H(1)	0.29085
H(1)	-0.37994
C(13)	-0.256
H(1)	0.03928
H(1)	0.03928
C(13)	2.64148
H(1)	-0.37995
H(1)	0.29092
C(13)	-7.05562
C(13)	2.91992
H(1)	-0.45011
H(1)	-0.6953
H(1)	-0.38955
C(13)	9.66808
H(1)	1.59701
H(1)	-1.02052
H(1)	-0.66146
C(13)	9.66797
H(1)	1.59699
H(1)	-1.02052
H(1)	-0.66147
C(13)	2.92004
H(1)	-0.45011
H(1)	-0.6953
H(1)	-0.38957

Table S22. The isotropic Fermi contact couplings in TEMPO calculated using the following approximation: uPBE1PBE/LanL2DZ prop=EPR nosymm int=ultrafine iop(3/75=7) SCRF=(Solvent= dimethylsulfoxide).

Atom	Gauss
N(14)	15.95935
O(17)	-27.41573
C(13)	-7.13015
C(13)	2.67955
H(1)	0.29332
H(1)	-0.40672
C(13)	-0.26291
H(1)	0.04082
H(1)	0.04082
C(13)	2.67957
H(1)	-0.40672
H(1)	0.29333
C(13)	-7.13016
C(13)	2.98268
H(1)	-0.46133
H(1)	-0.69744
H(1)	-0.40242
C(13)	9.83588
H(1)	1.67938
H(1)	-1.05468
H(1)	-0.69796
C(13)	9.83585
H(1)	1.67938
H(1)	-1.05468
H(1)	-0.69796
C(13)	2.9827
H(1)	-0.46133
H(1)	-0.69744
H(1)	-0.40242

Table S23. The isotropic Fermi contact couplings in TEMPO calculated using the following approximation: uPBE1PBE/LanL2DZ prop=EPR nosymm int=ultrafine iop(3/75=7) SCRF=(Solvent=n-hexane).

Atom	Gauss
N(14)	14.99604
O(17)	-28.30909
C(13)	-7.04083
C(13)	2.63324
H(1)	0.29083
H(1)	-0.37496
C(13)	-0.25473
H(1)	0.03898
H(1)	0.03898
C(13)	2.63329
H(1)	-0.37496
H(1)	0.29085
C(13)	-7.04084
C(13)	2.90898
H(1)	-0.44794
H(1)	-0.69499
H(1)	-0.38718
C(13)	9.63665
H(1)	1.58258
H(1)	-1.01435
H(1)	-0.65469
C(13)	9.63661
H(1)	1.58258
H(1)	-1.01435
H(1)	-0.65469
C(13)	2.90902
H(1)	-0.44794
H(1)	-0.69499
H(1)	-0.38719

Table S24. The isotropic Fermi contact couplings in 1:1 complex of TEMPO and water calculated using the following approximation: uPBE1PBE/LanL2DZ prop=EPR nosymm int=ultrafine iop(3/75=7) SCRF=(Solvent=water).

Atom	Gauss
N(14)	17.27367
O(17)	-26.14247
C(13)	-7.26584
C(13)	2.87959
H(1)	0.43733
H(1)	-0.45161
C(13)	-0.28052
H(1)	0.04228
H(1)	0.04335
C(13)	2.84732
H(1)	-0.45102
H(1)	0.38101
C(13)	-7.24401
C(13)	3.0651
H(1)	-0.49129
H(1)	-0.66072
H(1)	-0.44565
C(13)	10.05779
H(1)	1.74947
H(1)	-1.11600
H(1)	-0.79094
C(13)	10.52314
H(1)	1.87197
H(1)	-1.08079
H(1)	-0.78221
C(13)	2.95143
H(1)	-0.49185
H(1)	-0.70468
H(1)	-0.43959
O(8)	-0.87727
H(1)	-0.76131
H(1)	0.14183
